# Tissue-agnostic cancer drugs in the fight against molecular subsets of metastases of unknown origin

**DOI:** 10.18632/oncoscience.492

**Published:** 2019-10-04

**Authors:** Luca Roncati, Beniamino Palmieri

**Affiliations:** ^1^ Institute of Pathology, University Hospital of Modena, Policlinico, Modena, Italy

**Keywords:** metastasis of unknown origin (MUO), next generation sequencing (NGS), tissue-agnostic cancer drugs, pembrolizumab, larotrectinib

Metastasis of unknown origin (MUO) means that a cancer is detected when it is already in metastasis, but without any evidence of the primary tumor, even after a full clinico-radiological workup, histological examination, immunohistochemical investigations and tissue-of-origin testing. [1] The occult tumor cannot be identified because it has regressed, due to immune system, or because it is too small for imaging; however, it has been proposed that the undetectable size and the apparent dormant status do not preclude the spreading of cancer stem cells from the original tissue, giving rise to bulky mass at distant sites. [2] About 90% of MUOs are adenocarcinomas, the remaining squamous cell carcinomas, neuroendocrine tumors, composite malignancies and poorly differentiated or anaplastic neoplasms. [3] MUO occurs in about 3 to 5% of all oncological patients and it is burdened by a poor prognosis in 80-85% of the cases, due to its wide dissemination; the survival rates range from 6 to 16 months. These rates are lower in those patients with visceral involvement, ranging from 6 to 9 months. [3] Surely, to know the primary tumor is important because it dictates the best treatment plan and the expected outcome. However, thanks to next generation sequencing (NGS), it is nowadays possible to bypass this dogma and to search for driver mutations in hundreds of cancer-related genes with high sensibility and specificity in a 10-days turnaround time, by exploiting formalin-fixed paraffin-embedded metastatic bioptic samples or circulating tumor DNA in the patient's blood. [4] In addition to reveal the clinically actionable mutations for a personalized therapy, this modern technology provides information about the mutational burden and microsatellite instability (MSI). [4, 5] This last datum is particularly relevant because, on May 2017, the U.S. Food and Drug Administration (FDA) has approved the immunotherapy with pembrolizumab for all unresectable or metastatic, MSI-high (MSI-H) or mismatch repair deficient (dMMR), solid malignancies and, thus, pembrolizumab has become the first tissue-agnostic cancer drug. [6] Similarly, on November 2018, the FDA has approved the use of larotrectinib for all adult and pediatric solid tumors characterized by neurotrophic tyrosine receptor kinase (NTRK) gene fusion. [7] The adjective ‘agnostic’ (‘without knowledge’ from Ancient Greek) indicates a new subset of anti-cancer drugs (e.g. Keytruda^®^, Vitrakvi^®^) targeted towards the tumor on the basis of specific mutations found inside it, without knowing its tissue/site of origin just as happens in MUO. Therefore, the tissue-agnostic cancer drugs represent current treatment options against NTRK fusion-positive or MSI-H/dMMR MUOs. Other pipelines under development include: entrectinib for the therapy of pediatric and adult patients with recurrent or refractory extracranial solid tumors harboring NTRK, c-Ros oncogene 1 (ROS1) or anaplastic lymphoma kinase (ALK) gene fusions; BLU-667 and Loxo-292 for solid neoplasms with alterations in the “rearranged during transfection” (RET) gene; and Loxo-195 for NTRK fusion-positive solid cancers. [8–11] Preliminary clinical proof-of-principle data concerning with anti-human epidermal growth factor receptor 3 (Her3) antibodies in the treatment of solid malignancies with rearrangements in the neuregulin 1 (NRG1) gene have been already published. [12] All this testifies a great scientific attention around tissue-agnostic cancer drugs in an effort to provide more and more effective therapies to tumor bearer patients.
